# Identifying Public Response Topics to the Centers for Disease Control and Prevention’s COVID-19 Communications on Social Media: Infoveillance Study Using Large Language Model–Based Rephrasing

**DOI:** 10.2196/98319

**Published:** 2026-10-08

**Authors:** Wangjiaxuan Xin, Shuhua Yin, Albert Park, Yaorong Ge, Shi Chen

**Affiliations:** 1 College of Computing and Informatics, The University of North Carolina at Charlotte, 9201 University City Blvd, Charlotte, NC, 28223, United States; 2 Department of Public Health and Health Administration, The University of North Carolina at Charlotte, Charlotte, NC, 28223, United States; 3 School of Health Information Science, University of Victoria, Victoria, BC, V8W 2Y2, Canada; 4 School of Data Science, The University of North Carolina at Charlotte, Charlotte, NC, 28223, United States

**Keywords:** text mining, topic modeling, public health infoveillance, COVID-19, social media analytics, large language models

## Abstract

**Background:**

Public health agencies increasingly use social media platforms such as Twitter (subsequently rebranded X, X Corp) to monitor public responses to official health communications and support timely decision-making during health crises. Public replies to communications from official agencies provide valuable insights into population-level discourse and engagement with health policies. However, analyzing large-scale short-text responses remains challenging because social media replies are often brief, informal, ambiguous, and linguistically fragmented. Topic modeling offers a scalable approach for identifying thematic patterns in such data, but its performance is often limited when applied to social media short texts. Recent advances in large language models (LLMs) provide new opportunities to improve text normalization before topic modeling, but their value and limitations for public health infoveillance remain underexplored.


**Objective:**

This study develops and evaluates TM-Rephrase, a model-agnostic LLM-based rephrasing framework designed to improve the performance of topic models for short public health social media texts while ensuring rephrasing preserves the original semantic meaning, stance or intent, and tone.

**Methods:**

We analyzed 25,027 public replies to official Centers for Disease Control and Prevention tweets on Twitter collected between May 2020 and November 2022. TM-Rephrase transformed informal and noisy short texts into more standardized expressions using 2 prompt-guided schemes: general rephrasing and colloquial-to-formal rephrasing. Original and rephrased texts were analyzed using multiple topic models, with rephrasing generated by Gemini 2.5 Flash, GPT‑4o mini, and Mistral-7B-Instruct. Topic quality was evaluated using coherence, uniqueness, redundancy, and diversity. We also conducted an expert semantic-fidelity validation study in which 4 expert raters evaluated whether rephrased texts preserved the original meaning, stance or intent, and tone.

**Results:**

TM-Rephrase generally improved topic quality across models, rephrasing schemes, and LLM backbones. For latent Dirichlet allocation, $C_{v}$ coherence increased from 0.3094 without rephrasing to 0.5004 with colloquial-to-formal rephrasing. For BERTopic (BERT-based topic modeling), $C_{v}$ coherence improved from 0.4078 to 0.4734. Diversity-related metrics also improved in most rephrasing settings. Additional checks using $C_{NPMI}$, $C_{UCI}$, and $U_{MASS}$ generally supported the main improvement pattern. Sensitivity analysis across different topic numbers further suggested that the coherence gains were not limited to the main setting. Expert validation showed favorable semantic preservation overall, with a mean rating of 3.90 (SD 1.01) and median of 4.00 (IQR 3.00-5.00) of 5.00, and acceptable interrater reliability, intraclass correlation coefficient =0.736. General rephrasing received stronger semantic-fidelity ratings than colloquial-to-formal rephrasing, suggesting that stronger formalization may improve topic readability while introducing greater risk of changes to the original meaning, stance or intent, and tone.

**Conclusions:**

TM-Rephrase can enhance the coherence, distinctiveness, and interpretability of topic modeling outputs for short, noisy public health social media data. However, it is a text-normalization preprocessing strategy for topic enhancement rather than a fully neutral replacement for original public discourse.

## Introduction



### Background

The wide adoption of social media has fundamentally transformed public discourse, particularly during public health crises (eg, the COVID-19 pandemic) [1]. Twitter, characterized by rapid information dissemination and a concise conversational format, has emerged as a critical yet methodologically challenging data source for understanding and analyzing public discussion topics and the dynamics of information exchange during the COVID-19 pandemic [2]. In addition to enabling real-time communication, these platforms support large-scale public engagement, where individuals actively express opinions, share experiences, and respond to official health communications. For public health agencies such as the US Centers for Disease Control and Prevention (CDC), analyzing short-text public replies that express opinions and concerns provides valuable insights for informing more effective communication strategies to the public [3]. Such innovative surveillance and analyses can help capture evolving public concerns, assess responses to current health policies, and improve the alignment between institutional messaging and public needs. However, from a public health perspective, effectively leveraging these data remains challenging because of the unstructured nature of short, user-generated social media texts, which complicates the systematic monitoring and timely interpretation of population-level responses. In particular, the inherent brevity, informality, and ambiguity of individual tweets, along with the 280-character limit [4], pose significant challenges for traditional social media data mining and analytics. These characteristics are particularly problematic for topic modeling, which aims to identify latent thematic structures in textual corpora, because limited textual context constrains reliable semantic interpretation.

The contextual sparsity of social media short texts often leads to topics of keywords that are less coherent and less diverse (ie, more overlapping topics with redundant or unrelated keywords), thereby reducing interpretability and limiting their utility for understanding various public opinions during the pandemic. This limitation is further compounded by the heterogeneous nature of online public discourse, where variations in writing and expression styles introduce additional complexity into textual data. This problem becomes particularly pronounced when attempting to capture emerging and latent thematic structures in communications during public health emergencies, where semantic ambiguity, brevity, and informal linguistic styles (eg, abbreviations, slang, layman expressions, and misspellings) are prevalent [5]. Moreover, even when topic models identify clusters of topic keywords, they often lack meaningful interpretability without additional contextualization or external knowledge from experts, making it difficult to translate model outputs into coherent narratives for public health decision support. Consequently, these challenges limit the effectiveness of topic modeling in supporting systematic analysis of public health discourse. These issues suggest the need to standardize and formalize short social media texts into more contextually formal content before the application of topic modeling in health communications, thereby improving semantic clarity and enhancing the interpretability of downstream analytical results.

Topic modeling, as a widely used method for discovering latent thematic structures in textual corpora, is exemplified by the classic probabilistic model, latent Dirichlet allocation (LDA), proposed by Blei et al [6] in 2003. However, despite its broad adoption, applying LDA or its variants to short texts from social media platforms poses substantial challenges. The brevity, informality, and noise of tweets weaken statistical signals and exacerbate the context sparsity, leading to incoherent, redundant, or less interpretable topics generated from these probabilistic models [7].

To address these challenges, some enrichment strategies have been proposed. One line of research augments short texts with external knowledge or auxiliary contextual signals to enhance statistical robustness [8]. Another direction leverages distributed representations of words and documents, incorporating embeddings or neural architectures to map texts into richer semantic representations. Prominent examples include BERTopic (BERT-based topic modeling) [9] and Top2Vec [10], which use more recent transformer-based embeddings and clustering methods to generate more semantically meaningful topics. Recent work has explored more advanced neural network architectures, such as FASTopic (fast, adaptive, stable, and transferable topic model) [11] and topic-semantic contrastive learning [12], which demonstrate improved handling of context sparsity and have the potential to be applied in health communications.

However, all these approaches focus on algorithmic model redesign with parameterized inference strategies for short texts, which may adapt poorly to real-life large-scale data (eg, public health-related discourse on social media). In addition, such topic modeling algorithms typically output sets of top-ranked keywords that can be difficult for analysts or readers without additional domain knowledge to interpret.

Regarding the public discourse during COVID-19, the pandemic fundamentally transformed social media into the primary platform for real-time public discourse, where Twitter has been extensively used to study public attitudes and evolving concerns toward the pandemic and various health policies [13,14]. Scholars have analyzed public reactions to official communications from health agencies such as the CDC, focusing on opinion and thematic feedback to health policies [3,15]. Additional research has explored geospatial and demographic variations in public concerns, demonstrating the heterogeneous impact of the pandemic on different communities [16]. Despite the active research and the value of social media for effective public health infoveillance during emergencies, it often overlooks the interpretability issues arising from short and noisy texts in online discourse, especially on social media platforms.

Motivated by Maini et al [17], we leverage large language models (LLMs) as a text preprocessing approach for rephrasing short texts for topic modeling, aiming to standardize and formalize public replies to CDC tweets on Twitter. This framework, termed TM-Rephrase, builds on recent advances in LLMs, which have demonstrated powerful performance across diverse text-processing tasks, including text summarization [18], sentiment analysis [19], and information retrieval [20].

TM-Rephrase differs from conventional text preprocessing approaches. Standard text normalization and spelling correction primarily address surface-level noise, such as misspellings, inconsistent casing, punctuation, or informal abbreviations. Data augmentation usually creates additional training examples to improve model learning. In contrast, TM-Rephrase uses LLM-based, prompt-constrained rephrasing to transform each short social media reply into a more standardized and contextually explicit expression for topic modeling. Its distinguishing characteristic lies in treating rephrasing as a model-agnostic, data-centric preprocessing strategy for improving topic-model outputs rather than as a replacement for topic modeling algorithms or as generic text rewriting.

In this study, we examine the impact of TM-Rephrase on the quality of topics derived from 4 representative topic models, using a dataset of public replies to CDC Twitter tweets during the COVID-19 pandemic. We hypothesize that rephrasing original short-text public replies on social media through TM-Rephrase enhances topic quality in terms of both intratopic semantic coherence and inter–topic metrics of topic model outputs, which are represented as sets of keywords. Specifically, we investigate two rephrasing schemes: (1) general rephrasing and (2) colloquial-to-formal rephrasing, and evaluate their effectiveness across multiple topic modeling algorithms, including probabilistic algorithms (eg, LDA [6]) and more advanced neural network-based topic models (eg, BERTopic [9] and FASTopic [11]).

### Research Questions and Objectives

The research questions (RQs) of this study are as follows: (RQ1) whether and how effectively TM-Rephrase improves the quality and interpretability of topic model outputs for short texts in health communications, (RQ2) how different rephrasing schemes influence topic model performance, and (RQ3) how to interpret public discussions (ie, replies to CDC during the COVID-19 pandemic) based on the topics and inform public health practice.

This study aims to improve the analysis of public responses to official public health communications on social media to support more effective public health surveillance. To achieve this, we develop and evaluate TM-Rephrase, an LLM-based model-agnostic framework that enhances the quality and interpretability of topic modeling for short-text public replies to CDC communications during the COVID-19 pandemic.

## Methods

### Study Design

TM-Rephrase is designed as a multistage pipeline to improve topic modeling performance in public health communications, as shown in Figure 1. The pipeline is composed of four major stages: (1) data collection, (2) LLM-based rephrasing, (3) data preprocessing, and (4) topic modeling with a selected algorithm and evaluation of the resulting topics, as detailed in the following subsections. We used this pipeline to study how rephrased text of public responses to CDC tweets on Twitter impacts topic modeling outcomes compared to nonrephrased ones.



Before introducing the model details, we begin by formulating the problem setting with preliminary notations that are used throughout this paper. Let $D=\{d_{1},d_{2},\ldots ,d_{N}\}$ denote a corpus of *N* short-text tweets (ie, replies to CDC tweets). Each document $d_{i}$ is typically brief and context-limited because of the inherent sparsity of social media discourse.

The goal of topic modeling in this setting is to extract and model a set of *K* latent topics, denoted by $T=\left\{T_{1},T_{2},\ldots ,T_{K}\right\}$, where each topic $T_{k}$ is represented by a set of its top-*T* representative keywords, extracted or generated by the corresponding topic modeling algorithm, that is, $T_{k}=\left\{w_{1},w_{2},\ldots ,w_{T}\right\},k=1,2,\ldots ,K.$ These representative keywords serve as the semantic expression of the topic and form the basis for downstream topic interpretation, evaluation, and further analysis.

**Figure 1.**
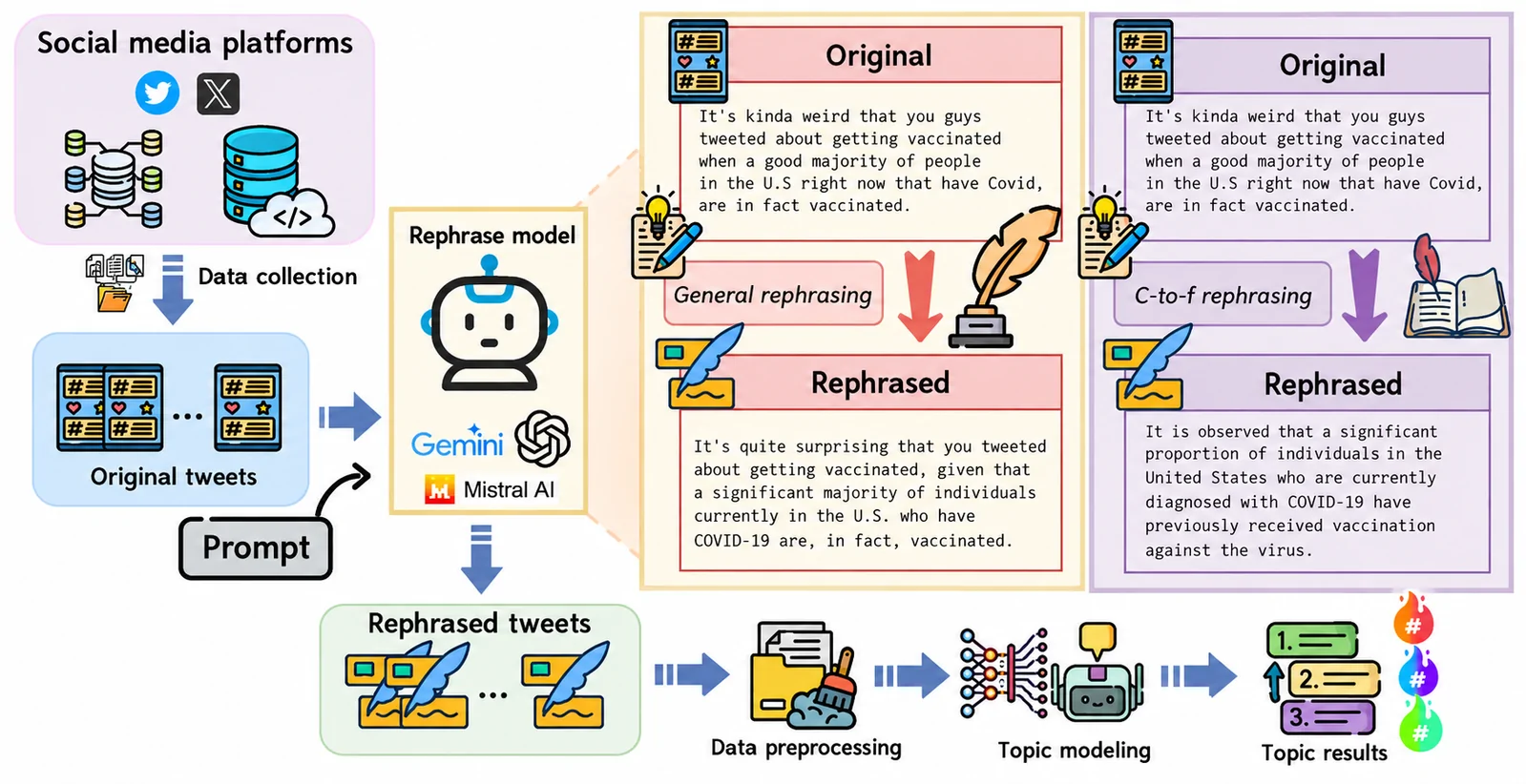
Overview of the TM-Rephrase framework. The pipeline integrates data collection, LLM–based rephrasing, data preprocessing, and topic modeling algorithm application with evaluation.

### Data Collection

We constructed a dataset of public responses to official CDC communications on Twitter during the COVID-19 pandemic, covering the period from May 2020 to November 2022. Data were collected via the Twitter Academic API and search queries (Section 1: data search queries in Multimedia Appendix 1), tracking 7 official CDC accounts: @CDCgov, @CDCDirector, @CDCGlobal, @CDCMMWR, @CDCtravel, @DrReddCDC, and @CDCEmergency. The initial retrieval yielded 180,090 public interactions associated with these CDC accounts, including replies and direct mentions. The retrieved metadata included tweet posting dates, tweet text, tweet account ID, account type, public engagement metrics, referenced tweet type, and referenced tweet IDs. Amazon Web Services was used as the computing or storage environment for managing the collected data. For the purpose of this study, only tweets that could be linked to an original CDC tweet were retained. Approximately 49,000 direct mentions that referenced a CDC account but were not replies to a specific CDC tweet were removed. For example, a tweet that mentions @CDCEmergency independently from a user’s own account was not considered a reply to the CDC communication. Another 104,000 reply-like interactions were further excluded as they could not be reliably linked to an accessible original CDC tweet, primarily because the original CDC tweet was deleted, unavailable, or otherwise inaccessible at the time of data processing. This step was necessary to preserve conversational context between each public reply and the corresponding CDC communication.

The final analytic dataset consisted of 25,027 public replies linked to 1,512 unique original CDC tweets. These replies capture public opinions, concerns, and discussions in response to official CDC communications during a major public health emergency. We retained only English-language tweets. Statistics of the final original and rephrased datasets are presented in Table 1.

**Table 1.** Statistics of the original and rephrased datasets of public replies to CDC^a^ communications during the COVID-19 pandemic.

Quantity of original and rephrased datasets	Original	General rephrased	C-to-f^b^ rephrased
Mean number of words	27.33	27.76	28.14
SD of word counts	14.23	15.12	14.11
Minimum number of words	1	1	1
25th percentile number of words	15	16	16
Number of words, median (IQR)	27 (15-40)	27 (16-41)	29 (16-42)
75th percentile number of words	40	41	42
Maximum number of words	64	66	68

a CDC: US Centers for Disease Control and Prevention.

b C-to-f: colloquial to formal.

Formally, the dataset $D=\{d_{1},d_{2},\ldots ,d_{N}\}$ denotes the original corpus, where each short-text reply is denoted as:


(1) $$d_{i}=\left(w_{1}^{i},\;w_{2}^{i},\;\ldots ,w_{L_{i}}^{i}\right),\;i=1,2,\ldots ,\;N$$

which contains a limited number of tokens $L_{i}$ of tweet $d_{i}$ as each tweet reply is limited to 280 characters [4]. The short and informal nature of these short texts results in sparse lexical co-occurrence signals, motivating the need for semantic enrichment before topic modeling algorithms are applied.

### LLM-Based Rephrasing

The second stage introduces an LLM-based rephrasing operator that transforms each short-text document (ie, a public reply to a CDC tweet) into a semantically and contextually refined version that is similar to CDC’s official communication. Let $R_{\theta }$ denote a rephrase model (ie, an LLM) parameterized by *θ*. For each short-text reply $d_{i}$, the rephrasing process is defined as:


(2) $${\hat{d}}_{i}=R_{\theta }(\pi ,d_{i}),\;i=1,2,\ldots ,N$$

where $\hat{d_{i}}$ refers to the rephrased short-text reply, and π denotes a carefully designed prompt that constrains the generation process to preserve semantic fidelity and ensure that rephrasing is executed under a certain scheme that will be introduced in this subsection. This rephrasing stage is crucial for the downstream topic modeling of the public replies and for better understanding of public responses to official health communications during emergencies.

For the rephrasing stage, we experimented with 3 different LLMs: Google Gemini (Gemini 2.5 Flash [21]), OpenAI generative pretrained transformer (GPT‑4o mini [22]), and an open-source LLM (Mistral-7B-Instruct [23]), given their strong natural language generation capabilities and complementary trade-offs between performance and cost for large-scale applications. We used a low sampling temperature (0.2) to ensure semantic fidelity and minimize stochastic variation during automated rephrasing, consistent with findings from prior work [24].

To systematically investigate how different styles of linguistic refinement influence topic modeling performance, TM-Rephrase incorporates 2 distinct prompt-guided rephrasing schemes that we developed: general rephrasing and colloquial-to-formal rephrasing. Although both schemes are designed to preserve semantic fidelity, they differ in the degree and style of linguistic transformation applied to the original short texts of public replies. Prompts for these 2 schemes are shown in Table S1 of Multimedia Appendix 1 in Section 2: prompt information for rephrasing schemes.

The general rephrasing scheme performs lightweight linguistic refinement while strictly maintaining the meaning and informational content of the original text. Its primary objective is to improve grammatical correctness, syntactic clarity, and overall readability without altering domain-specific terminology, named entities, hashtags, usernames, or technical details. Concretely, the general rephrasing scheme fixes typographical errors, incomplete sentence fragments, inconsistent verb tenses, and ambiguous phrasing, all of which are common in social media discourse. It may restructure fragmented clauses into complete sentences and resolve minor ambiguities that arise from informal writing conventions. However, it avoids introducing new concepts, removing key terms, or substituting specialized vocabulary. As such, general rephrasing can be viewed as a minimal-intervention normalization process that enhances structural regularity while preserving the lexical distribution of domain-relevant tokens.

The colloquial-to-formal rephrasing scheme transforms informal, conversational, or slang-based expressions into formal, professional English suitable for public health communication contexts. The rephrased outputs are structured to resemble the tone and style of public health reports or professional summaries, using complete sentences and standardized grammar while avoiding slang, contractions, and casual phrasing. During this process, implicit references—such as omitted subjects, abbreviated expressions, or context-dependent meanings (eg, “this shot,” “they said,” or fragmentary statements lacking clear referents)—are made explicit by clarifying the intended subject, action, or relationship within the sentence. Fragmented or elliptical constructions are rewritten into fully articulated and logically coherent statements to improve readability and interpretability. Strict constraints are applied to preserve the original meaning, including retaining all named entities, hashtags, usernames, and domain-specific terminology. No additional claims, interpretations, or external information are introduced.

These two rephrasing schemes enable a controlled comparison between moderate linguistic normalization and more substantial stylistic formalization for health communications. This design allows us to evaluate whether incremental grammatical refinement alone suffices to improve topic modeling performance, or whether deeper formalization is required for optimal topic quality. In order to ensure semantic alignment between the original and rephrased texts, the prompt explicitly instructs the LLMs to retain meaning and avoid modifying domain-specific content.

The resulting rephrased corpus is defined in equation 3, where $\hat{d_{i}}$ represents each individual rephrased short-text reply:


(3) $$\hat{D}=\{{\hat{d}}_{1},{\hat{d}}_{2},\ldots ,{\hat{d}}_{N}\}$$

### Expert Validation of Semantic Fidelity

To evaluate whether LLM-based rephrasing preserved the meaning, stance or intent, and tone of the original public replies, we further conducted a human expert validation study. A sample of 30 original CDC-reply tweets was randomly selected for validation. For each original tweet, 2 rephrased versions were evaluated: one generated using the general rephrasing scheme and one generated using the colloquial-to-formal scheme, resulting in 60 original-rephrased text pairs across 2 schemes.

Four expert raters with complementary expertise in health communication, social media research, linguistics, and public health independently evaluated the rephrased texts. Each rater compared the original tweet with the corresponding rephrased version in a paired format and provided a single holistic semantic-fidelity rating using a 5-point Likert scale. Raters were instructed to consider whether the rephrased version preserved the original meaning, stance or intent, and tone, but these dimensions were not rated separately. As the task required direct comparison between original and rephrased texts, complete blinding to the rephrasing scheme was not feasible. Raters completed the evaluations independently. We summarized the validation results using the mean, median, and distribution of expert ratings. To assess interrater reliability, we calculated a 2-way random-effects, absolute-agreement intraclass correlation coefficient (ICC[2,4]) for the aggregated expert ratings.

### Data Preprocessing

Before applying topic modeling, both the original and rephrased corpora undergo a consistent and standardized preprocessing procedure to ensure comparability and reduce extraneous noise. The preprocessing step includes the removal of URLs, punctuation marks, and stop words, which do not contribute meaningful semantic information to topic inference during topic modeling.

At the same time, semantically meaningful elements are carefully preserved. Emojis are retained through their textual descriptions to maintain their affective or contextual signals. For example, the emoji 😊 is converted to a textual representation “smiling face,” and 😷 is mapped to “face with medical mask.” This conversion ensures that nontextual, yet informative elements remain encoded within the lexical space rather than be discarded as noise.

All texts are further normalized by converting all characters to lowercase, followed by tokenization and lemmatization. Lowercasing eliminates case-based lexical duplication, tokenization segments text into analyzable units, and lemmatization reduces inflected forms of tokens to their base form, thereby consolidating semantically equivalent variants into unified lexical representations. All these operations promote lexical consistency and stabilize downstream word co-occurrence statistics in topic modeling.

Formally, let $P_{\gamma }\left(∙\right)$ denote the preprocessing operator parameterized by γ, which encapsulates the sequence of normalization, tokenization, and lemmatization in the data preprocessing step. After preprocessing, each original document $d_{i}$ and rephrased document $\hat{d_{i}}$ are respectively converted into the preprocessed format:


(4) $$p_{i}=P_{y}(d_{i}),\;{\hat{p}}_{i}=P_{y}({\hat{d}}_{i}),\;i=1,2,\ldots ,N$$

Here $p_{i}\in R^{\left|V\right|}$ and $\hat{p_{i}}\in R^{\left|V\right|}$ denote the tokenized representations of the original and the rephrased documents (ie, short-text replies), respectively, under a shared vocabulary space *V*. Maintaining identical preprocessing across both corpora ensures that any observed differences in topic modeling performance can be credited to the rephrasing rather than discrepancies in preprocessing or representation learning.

### Topic Models

In the final stage of the pipeline, topic modeling algorithms are applied, and their performance is systematically evaluated. As illustrated in Figure 2, both the original and rephrased replies are modeled by the same topic models, and the resulting topics are compared and evaluated through quantitative metrics and expert semantic fidelity evaluation. The original corpus D and rephrased corpus $\hat{D}$ are independently input into the same specific topic modeling algorithm $M_{TM}$ to ensure methodological consistency:


(5) $$T=M_{TM}(D),\;\hat{T}=M_{TM}(\hat{D})$$

This parallel modeling design yields 2 corresponding sets of topic outputs, that is, one derived from the original texts and the other from the rephrased texts. This enables a direct and controlled comparison. Due to the same topic modeling procedure, hyperparameter settings for topic models and preprocessing configurations are maintained across both corpora. Therefore, any observed differences in topic quality can be credited to the rephrasing scheme rather than the specific topic modeling algorithm.

To comprehensively evaluate the effects of TM-Rephrase, 4 representative topic models were applied, including conventional statistical and neural network-based topic models. This suite of different topic models enables a systematic analysis of how different topic modeling algorithms respond to TM-Rephrase.

**Figure 2.**
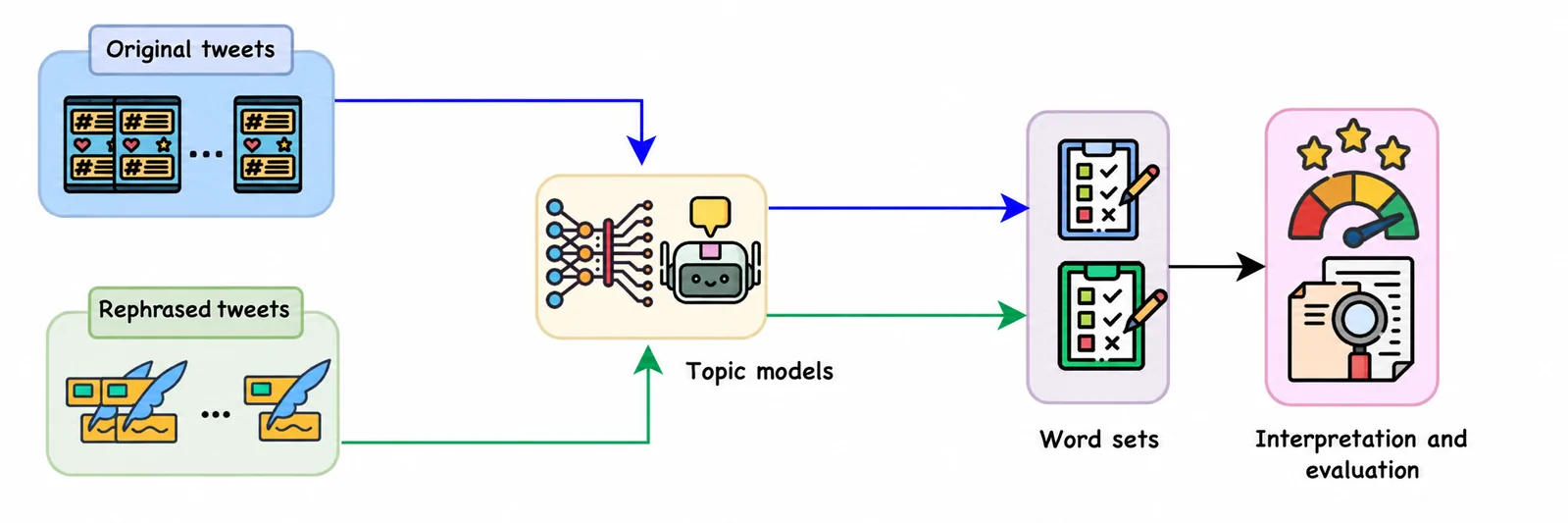
Outline of the topic evaluation process. Both the original and rephrased tweets were modeled using the same topic models, and the resulting topics were compared and evaluated through quantitative metrics and expert semantic fidelity evaluation.

LDA [6] is a foundational probabilistic topic model serving as the benchmark in topic modeling studies. It is effective for classic topic discovery but is usually challenged by data sparsity in short social media texts.

BERTopic [9] is a neural topic model using contextual embeddings (eg, Sentence-BERT [25]) and density-based clustering. It is considered well-suited for noisy, informal social media data.

FASTopic [11] is based on a transformer backbone that directly captures semantic relationships between document embeddings and learnable topic-word embeddings. It uses an embedding transport plan as an optimization objective to enhance topic-word and document-topic associations.

Topic-semantic contrastive topic model (TSCTM) [12] addresses short-text sparsity via contrastive learning, generating dense semantic vectorized representations and topic distributions.

Together, these different topic models ensure that the evaluation of TM-Rephrase is not limited to a single topic modeling paradigm, but captures methodological robustness across multiple topic models. For all 4 topic models, we fixed the number of topics (*K*) at 8, based on an exploratory analysis to balance thematic detail and interpretability for the study dataset. For each topic, represented as a set of keywords, we retained the top 15 keywords , ranked according to the model’s topic-word probability. Details of the experimental implementation are available in Section S3: implementation details in Multimedia Appendix 1.

### Topic Modeling Performance Evaluation

#### Overview

Four quantitative evaluation metrics were computed to quantify topic coherence, uniqueness, redundancy, and diversity, with details shown below.

#### *Topic Coherence* ($C_{v}$)

$C_{v}$ [26] measures the semantic coherence among the topic keywords, which are determined by topic-word probabilities. This metric was chosen based on the mathematical analysis and justifications in the study by Wu [27]. This metric fundamentally relies on normalized pointwise mutual information (NPMI) to quantify pairwise word semantic associations. For any 2 words $w_{i}$ and $w_{j}$, their NPMI score is defined as:


(6) $$NPMI\left(w_{i},\;w_{j}\right)=\frac{log\left(\frac{P\left(w_{i},\;w_{j}\right)+\epsilon }{P\left(w_{i}\right)P\left(w_{j}\right)+\epsilon }\right)}{-log\left(P\left(w_{i},\;w_{j}\right)+\epsilon \right)}$$

Here, $P\left(w_{i}\right)$ and $P\left(w_{j}\right)$ are the probabilities of observing words $w_{i}$ and $w_{j}$, respectively, and $P\left(w_{i},w_{j}\right)$ is the co-occurrence probability of the word pair $\left(w_{i},w_{j}\right)$ within a defined external corpus (the Wikipedia [Wikimedia Foundation, Inc] corpus was used in this study [28]). A small constant ϵ (eg, $10^{-12}$) is added to avoid division by 0 or taking the logarithm of 0. Building on the NPMI formulation, the $C_{v}$ coherence score can be formally expressed as follows:


(7) $$C_{v}=\frac{1}{T}\sum_{i=1}^{T}\cos\left(v_{NPMI}\left(x_{i}\right),\;v_{NPMI}\left({\left\{x_{j}\right\}}_{j=1}^{T}\right)\right)$$

Here, $v_{NPMI}\left(x_{i}\right)$ represents the vector of NPMI scores between a word $x_{i}$ and all other words in the topic keyword list, defined as:


(8) $$v_{NPMI}\left(x_{i}\right)={\left\{NPMI\left(x_{i},\;x_{j}\right)\right\}}_{j=1,\ldots ,T}$$

Similarly, the aggregated NPMI vector across all words in the topic keyword list is defined as:


(9) $$v_{NPMI}\left({\left\{x_{j}\right\}}_{j=1}^{T}\right)={\left\{\sum_{i=1}^{T}NPMI\left(x_{i},\;x_{j}\right)\right\}}_{j=1,\ldots ,T}$$

where *T* is the number of top keywords in the topic (*T*=15 in this study). The cosine similarity is then computed between the word-specific and aggregated vectors, and the final $C_{v}$ score is obtained by averaging across all words. A higher $C_{v}$ score indicates stronger semantic coherence and better interpretability of the topics.

#### Topic Uniqueness

Nan et al [29] proposed topic uniqueness (*TU*), a metric that quantifies how distinct the top keywords within each topic are across the entire topic set T. This helps identify whether individual topics use distinctive vocabulary. Given *K* topics and the top *T* keywords of each topic, *TU* is computed as:


(10) $$TU=\frac{1}{K}\sum_{k=1}^{K}\left(\frac{1}{T}\sum_{x_{i}\in T_{k}}\frac{1}{\#\left(x_{i}\right)}\right)$$

where $T_{k}$ denotes the top keyword set of the *k*-th topic, and $\#\left(x_{i}\right)$ denotes the occurrence count of word $x_{i}$ in the top *T* words of all topics. *TU* ranges from *1* /K to 1, and a higher *TU* score indicates more unique topics.

#### Topic Redundancy

Topic redundancy (*TR*) [30] was developed to measure the overlap of top keywords across different topics. The formula for *TR* is given as:


(11) $$TR=\frac{1}{K}\sum_{k=1}^{K}\left(\frac{1}{T}\sum_{x_{i}\in T_{k}}\frac{\#\left(x_{i}\right)-1}{K-1}\right)$$

A lower *TR* score suggests that the topics are more distinct.

#### Topic Diversity

The formula for the topic diversity *(TD)* [31], which measures the proportion of unique top keywords across all topics, is defined as:


(12) $$TD=\frac{1}{K}\sum_{k=1}^{K}\frac{1}{T}\sum_{x_{i}\in T_{k}}I\left(\#\left(x_{i}\right)\right)$$

A higher *TD* score indicates more diverse topics with less word overlap. This metric works by identifying words that are unique to a single topic’s top keyword list. The indicator function I(∙) is defined by a stepwise function. This function returns 1 if and only if a word $x_{i}$ appears in the top keyword list of exactly 1 topic, and 0 otherwise.

These 4 metrics, $C_{v}$, *TU*, *TR,* and *TD*, evaluate topic quality across complementary dimensions. In brief, $C_{v}$ measures semantic coherence among a topic’s top keywords, *TU* assesses topic uniqueness across topics, *TR* quantifies topic redundancy due to topical overlap, and *TD* evaluates whether the model produces a diverse set of topics. These metrics provide a comprehensive set of measures for evaluating and comparing topics generated from original nonrephrased tweets and those rephrased through 2 different TM-Rephrase schemes (general and colloquial-to-formal rephrasing).

Through systematic comparisons between generated topics based on original and rephrased texts respectively, we evaluated the extent to which the model-agnostic TM-Rephrase framework improves the intratopic coherence and intertopic quality.

### Ethical Considerations

This study analyzed publicly accessible online tweets from Twitter that were replies to official CDC communications during the COVID-19 pandemic. To comply with the platform’s privacy policy, data collection was limited to publicly accessible tweets. The social media data component did not involve direct interaction with Twitter users, recruitment of social media users, intervention, or collection of private information beyond publicly available tweet content. Informed consent from social media users was not obtained because the analysis was based solely on publicly accessible online tweets.

This study, including the human expert validation component, was reviewed by the Office of Research Protections and Integrity at the University of North Carolina at Charlotte and received a Notice of Determination of Exemption. This study was determined to meet the exempt category cited under 45 CFR 46.104(d), Exemption Category 2. The institutional review board study number is IRB-26‐1225, titled “Human Evaluation of Topic Modeling Performance for Text Analysis of Public Health-Related Social Media Short Texts,” with an approval date of July 8, 2026 (Figure S1 in Multimedia Appendix 1 in Section S4: notice of determination of exemption from IRB Office in UNC Charlotte).

To minimize privacy risks, no usernames, profile information, or other direct user identifiers are reported in this paper. Example tweets, when presented, are used only for methodological illustration, and the main findings are reported in aggregate form. Expert validation results are also summarized in aggregate, without identifying individual raters.

## Results

### Overview

This section presents the results of quantitative evaluation of TM-Rephrase, followed by illustrative examples. We report results across four topic models, FASTopic, BERTopic, TSCTM, and LDA, under three conditions, as follows: (1) original tweets without any rephrasing, (2) general rephrasing, and (3) colloquial-to-formal rephrasing, with three different LLMs used for rephrasing (ie, Gemini 2.5 Flash, GPT‑4o mini, and Mistral-7B-Instruct). Topic quality was quantitatively assessed using 4 metrics: $C_{v}$, *TU*, *TR*, and *TD,* as described in the Topic Modeling Performance Evaluation section.



Before introducing the quantitative results, we refer to Table 1 to assess potential text expansion or compression caused by TM-Rephrase. Both general and colloquial-to-formal rephrasing schemes hardly change token lengths: the mean increases marginally from 27.33 (original, SD 14.23) to 27.76 (general, SD 15.12) and 28.14 (colloquial-to-formal, SD 14.11), and the median changes slightly from 27 (IQR 15-40) to 29 (colloquial-to-formal, IQR 16-42). Quartiles and maxima show similar minor differences, indicating that rephrasing preserves the overall text length distribution, and any effects on topic modeling are unlikely to stem from token counts.

### Quantitative Results

#### Overview

Table 2 reports the quantitative evaluation results under a 3-factor design, including 4 topic modeling algorithms, 3 LLM backends, and 2 rephrasing schemes plus the original baseline, resulting in a total of 28 configurations. This design enables a systematic assessment of the main effects of rephrasing, as well as its interaction with model architecture and LLM choice.

**Table 2.** Quantitative metric results for various topic models, both with and without TM-Rephrase by three LLMs^a^^,^^b^.

	$C_{v}$(0‐1) ↑^c^	*TU^d^* (0.125‐1) ↑	*TR^e^* (0‐1) ↓	*TD^f^* (0‐1) ↑
FASTopic^g^ w/o^h^ rephr^i^^,^^j^	.3388	.9917	.0024	.975
Gemini 2.5 Flash				
FASTopic w/^k^ general rephr^j^	.3723	.9917	.0024	.9917
FASTopic w/ c-to-f rephr^j^	.3301	1^l^	0^l^	1^l^
GPT‑4o mini				
FASTopic w/ general rephr	.3746^l^	.9917	.0024	.9917
FASTopic w/ c-to-f rephr	.3422	1^l^	0^l^	1^l^
Mistral-7B-Instruct				
FASTopic w/ general rephr	.3728	.9917	.0024	.9917
FASTopic w/ c-to-f rephr	.3321	.9917	.0024	.9917
BERTopic^m^ w/o rephr	.4078	.4667	.3976	.4667
Gemini 2.5 Flash				
BERTopic w/ general rephr	.4564	.525^l^	.3357^l^	.525^l^
BERTopic w/ c-to-f rephr	.4734^l^	.525^l^	.3452	.525^l^
GPT‑4o mini				
BERTopic w/ general rephr	.4612	.4667	.3357^l^	.4667
BERTopic w/ c-to-f rephr	.4687	.525^l^	.3452	.525^l^
Mistral-7B-Instruct				
BERTopic w/ general rephr	.4574	.4667	.3357^l^	.4667
BERTopic w/ c-to-f rephr	.4713	.4667	.3976	.4667
TSCTM^n^ w/o rephr	.3094	.9917	.0024	.975
Gemini 2.5 Flash				
TSCTM w/ general rephr	.3145	.9833	.0048	.9833
TSCTM w/ c-to-f rephr	.3394^l^	1^l^	0^l^	1^l^
GPT‑4o mini				
TSCTM w/ general rephr	.3267	.9917	.0024	.9917
TSCTM w/ c-to-f rephr	.3331	1^l^	0^l^	1^l^
Mistral-7B-Instruct				
TSCTM w/ general rephr	.3104	.9833	.0024	.9833
TSCTM w/ c-to-f rephr	.3213	.9917	.0024	.975
LDA^o^ w/o rephr	.3094	.575^l^	.3095	.575^l^
Gemini 2.5 Flash				
LDA w/ general rephr	.4206	.5583	.3214	.5583
LDA w/ c-to-f rephr	.5004^l^	.575^l^	.3048^l^	.575^l^
GPT‑4o mini				
LDA w/ general rephr	.4197	.5583	.3214	.5583
LDA w/ c-to-f rephr	.4917	.575^l^	.3095	.575^l^
Mistral-7B-Instruct				
LDA w/ general rephr	.4008	.5583	.3095	.5583
LDA w/ c-to-f rephr	.4762	.5583	.3095	.5583

a LLM: large language model.

b Table rows of TM-Rephrase are results based on Gemini 2.5 Flash, GPT‑4o mini, and Mistral-7B-Instruct, respectively.

c Arrows in the header indicate the desired direction for each metric (higher ↑ or lower ↓ is better).

d TU: topic uniqueness.

e TR: topic redundancy.

f TD: topic diversity.

g FASTopic: fast, adaptive, stable, and transferable topic model.

h w/o: without.

i Rephr stands for the rephrasing.

j General rephr stands for the general rephrasing scheme, while c-to-f rephr stands for the colloquial-to-formal rephrasing scheme.

k w: with.

l The best results within each model group.

m BERTopic: BERT-based topic modeling.

n TSCTM: topic-semantic contrastive topic model.

o LDA: latent Dirichlet allocation.

#### Effect of Rephrasing Schemes

Across most configurations, TM-Rephrase improves topic quality, with the most pronounced and stable gains observed in topic coherence ($C_{v}$). However, the magnitude and consistency of improvement vary across topic models, evaluation metrics, and rephrasing schemes. In particular, the colloquial-to-formal rephrasing scheme generally yields the highest coherence scores. For example, with LDA, coherence increases substantially from 0.3094 (without rephrasing) to 0.5004 under Gemini 2.5 Flash, with similarly large improvements observed for GPT‑4o mini (0.4917) and Mistral-7B-Instruct (0.4762). These results indicate that rephrasing effectively mitigates contextual sparsity in short texts and enhances semantic consistency across topics.

As $C_{v}$ coherence relies on word co-occurrence statistics derived from an external reference corpus, its values may be influenced by how closely the topic keywords align with the lexical patterns of that corpus. In this study, the reference corpus was Wikipedia, which may better represent standardized or encyclopedic expressions than informal social media language. Therefore, colloquial-to-formal rephrasing may partly improve $C_{v}$ by shifting topic keywords toward lexical patterns more characteristic of Wikipedia, rather than solely by improving the substantive representation of public meaning.

#### Effect Across Topic Models

The magnitude of improvement varies across topic models. Probabilistic models such as LDA exhibit the largest gains, reflecting their strong dependence on lexical co-occurrence patterns. BERTopic shows consistent but more moderate improvements, with coherence increasing across all LLMs and rephrasing schemes, and reaching its highest value under Gemini 2.5 Flash ($C_{v}$=0.4734). In contrast, FASTopic demonstrates relatively smaller and less consistent gains, with slight decreases in coherence under the colloquial-to-formal scheme in some cases (eg, $C_{v}$=0.3301 under Gemini 2.5 Flash). This suggests that embedding-based models may already partially capture semantic relationships despite informal language, reducing the effectiveness of input standardization. TSCTM, meanwhile, benefits substantially from rephrasing in terms of diversity-related metrics, achieving perfect topic separation (*TU*=1, *TD*=1, and *TR*=0) under colloquial-to-formal rephrasing with both Gemini 2.5 Flash and GPT‑4o mini.

#### Effect of LLM Backbones

The improvements from TM-Rephrase are broadly similar across all 3 LLM backbones. Although minor variations in absolute performance are observed, the patterns remain clear and consistent: rephrasing improves coherence and diversity metrics across topic models regardless of the underlying LLM. For example, LDA coherence gains and TSCTM diversity improvements are observed consistently across Gemini 2.5 Flash, GPT‑4o mini, and Mistral-7B-Instruct. This stability suggests that the effectiveness of TM-Rephrase is not dependent on a specific LLM, but rather reflects a generalizable rephrasing strategy.

#### Diversity and Redundancy Metrics

In addition to improving intratopic coherence, rephrasing also improves intertopic quality. Across FASTopic, BERTopic, and TSCTM, *TU* and *TD* generally increase, while *TR* decreases or remains similar. The most notable improvement is observed in TSCTM under colloquial-to-formal rephrasing, where perfect diversity scores are achieved, indicating fully distinct topic clusters. These findings suggest that rephrasing enhances not only intratopic coherence but also intertopic separation.

Figure 3 shows that colloquial-to-formal rephrasing often achieves larger coherence gains than general rephrasing for LDA and BERTopic, while the overall improvement patterns are observed across Gemini 2.5 Flash, GPT‑4o mini, and Mistral-7B-Instruct. These results demonstrate the robustness of the TM-Rephrase framework as well.

**Figure 3.**
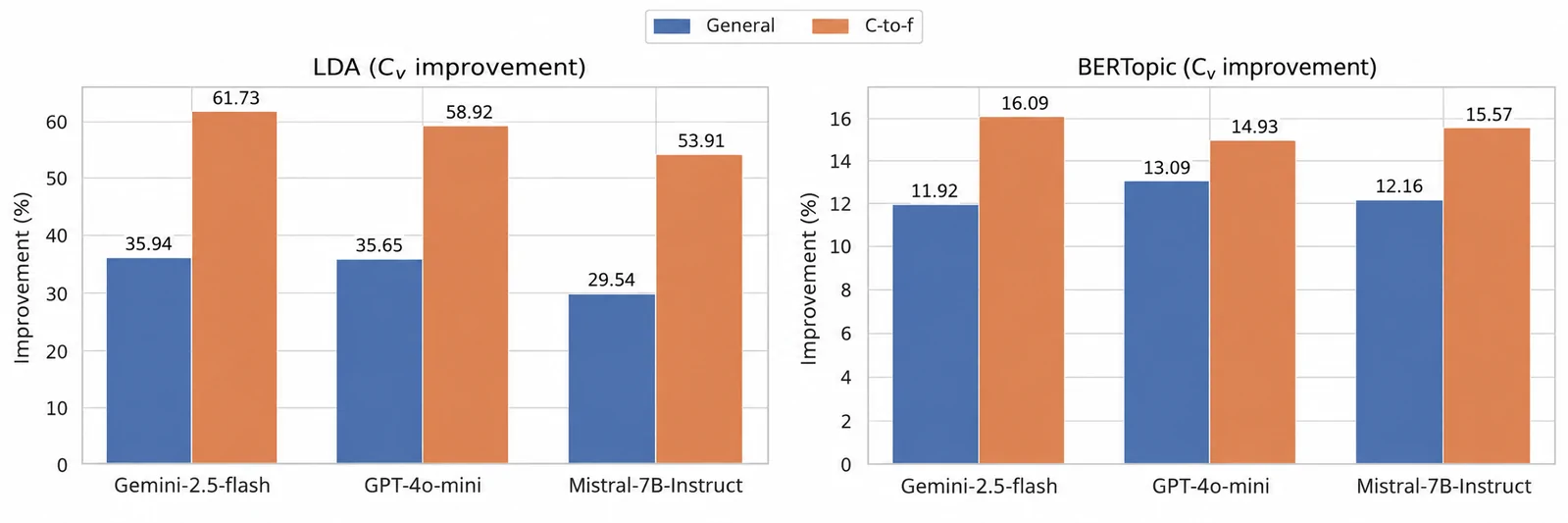
$C_{v}$ improvement (%) across large language models (LLMs) and rephrasing schemes for LDA and BERTopic. LDA: latent Dirichlet allocation.

Additionally, a nuanced trade-off between intratopic coherence and inter-*TD* emerges, especially for the colloquial-to-formal rephrasing scheme. While this scheme often delivers the highest coherence and perfect diversity scores, it can occasionally reduce *TU* in certain topic models. For example, colloquial-to-formal rephrasing maximizes coherence across all LLMs but slightly lowers *TU* compared to the nonrephrased baseline in LDA. This suggests that aggressive formalization might homogenize lexical choices across topics, highlighting the importance of matching rephrasing strength with model characteristics. In addition, the comparison across LLM backbones reveals a high degree of robustness in TM-Rephrase. While Gemini 2.5 Flash often achieves the strongest absolute results, GPT‑4o mini and Mistral-7B-Instruct closely track its performance across all metrics and models. The relative ranking of rephrasing schemes and topic models remains largely unchanged, indicating that TM-Rephrase is not tightly tied to a specific LLM but instead constitutes a generalizable topic-model-agnostic paradigm.

To demonstrate whether the observed improvements were dependent on the use of $C_{v}$ coherence alone, we further evaluated topic coherence using 3 additional metrics: $C_{NPMI}$ [26], $C_{UCI}$ [32], and $U_{MASS}$ [33]. These metrics provide complementary views of topic coherence based on word co-occurrence patterns, with higher values indicating better coherence for all 3 metrics. The results are reported in Table 3.

**Table 3.** Quantitative coherence metric results for various topic models, both with and without TM-Rephrase by 3 LLMs^a^^,^^b^.

	$C_{NPMI}$ (-1, 1) ↑^c^	$C_{UCI}$ (-∞, +∞) ↑	$U_{MASS}$ (-∞, 0] ↑
FASTopic^d^ w/o^e^ rephr^f^	−0.033	−1.477	−5.035
Gemini 2.5 Flash			
FASTopic w/^g^ general rephr^h^	−0.021^i^	−1.461	−4.671^i^
FASTopic w/ c-to-f rephr^h^	−0.036	−1.456^i^	−4.818
GPT‑4o mini			
FASTopic w/ general rephr	−0.027	−1.471	−4.833
FASTopic w/ c-to-f rephr	−0.031	−1.466	−4.874
Mistral-7B-Instruct			
FASTopic w/ general rephr	−0.024	−1.469	−4.726
FASTopic w/ c-to-f rephr	−0.035	−1.465	−4.819
BERTopic^j^ w/o rephr	.028	.38	−2.358
Gemini 2.5 Flash			
BERTopic w/ general rephr	.049^i^	.401	−2.18
BERTopic w/ c-to-f rephr	.045	.386	−2.163^i^
GPT‑4o mini			
BERTopic w/ general rephr	.031	.411^i^	−2.212
BERTopic w/ c-to-f rephr	.042	.387	−2.319
Mistral-7B-Instruct			
BERTopic w/ general rephr	.044	.385	−2.338
BERTopic w/ c-to-f rephr	.03	.381	−2.314
TSCTM^k^ w/o rephr	−0.021	−1.065	−3.512
Gemini 2.5 Flash			
TSCTM w/ general rephr	−0.017	−0.955	−3.141
TSCTM w/ c-to-f rephr	−0.013^i^	−0.876^i^	−2.949
GPT‑4o mini			
TSCTM w/ general rephr	−0.015	−1.032	−2.941^i^
TSCTM w/ c-to-f rephr	−0.016	−0.944	−2.997
Mistral-7B-Instruct			
TSCTM w/ general rephr	−0.02	−0.912	−3.217
TSCTM w/ c-to-f rephr	−0.018	−0.998	−3.111
LDA^l^ w/o rephr	.017	−0.084	−2.457
Gemini 2.5 Flash			
LDA w/ general rephr	.038	.282	−2.401
LDA w/ c-to-f rephr	.071^i^	.848^i^	−2.146
GPT‑4o mini			
LDA w/ general rephr	.069	.193	−2.39
LDA w/ c-to-f rephr	.058	.764	−2.418
Mistral-7B-Instruct			
LDA w/ general rephr	.047	.242	−2.06^i^
LDA w/ c-to-f rephr	.066	.656	−2.279

a LLM: large language model.

b Table rows of TM-Rephrase are results based on Gemini 2.5 Flash, GPT‑4o mini, and Mistral-7B-Instruct, respectively.

c Arrows in the header indicate the desired direction for each metric (higher ↑ or lower ↓ is better).

d FASTopic: fast, adaptive, stable, and transferable topic model.

e w/o: without.

f Rephr stands for rephrasing.

g w/: with.

h General rephr stands for the general rephrasing scheme, while c-to-f rephr stands for the colloquial-to-formal rephrasing scheme.

i The best results within each model group.

j BERTopic: BERT-based topic modeling.

k TSCTM: topic-semantic contrastive topic model.

l LDA: latent Dirichlet allocation.

The results support the main finding that TM-Rephrase generally improves topic coherence, although the magnitude of improvement varies across topic models, LLM backbones, and rephrasing schemes. The greatest and most consistent improvements are observed for LDA. For example, under Gemini 2.5 Flash, LDA improves from 0.017 to 0.071 on $C_{NPMI}$, from −0.084 to 0.848 on $C_{UCI}$, and from −2.457 to −2.146 on $U_{MASS}$ after colloquial-to-formal rephrasing. Similar improvements are also observed for GPT‑4o mini and Mistral-7B-Instruct, suggesting that the coherence gains for LDA are not specific to a single LLM backbone.

For BERTopic and TSCTM, the results also show improved coherence after rephrasing, although the best-performing rephrasing scheme differs by metric and LLM. For BERTopic, rephrased texts improve $C_{NPMI}$ and $C_{UCI}$ in most settings, with Gemini 2.5 Flash general rephrasing achieving the highest $C_{NPMI}$ score and GPT‑4o mini general rephrasing achieving the highest $C_{UCI}$ score. For TSCTM, both general and colloquial-to-formal rephrasing improve coherence relative to the baseline without rephrasing across most metrics, with colloquial-to-formal rephrasing under Gemini 2.5 Flash performing best on $C_{NPMI}$ and $C_{UCI}$.

The effects are less consistent for FASTopic. General rephrasing improves $C_{NPMI}$ and $U_{MASS}$ in several settings, while colloquial-to-formal rephrasing does not consistently improve $C_{NPMI}$ relative to the nonrephrased baseline. This suggests that embedding-based or neural topic models may already capture some semantic relationships in noisy short texts, making them less sensitive to input-level formalization than LDA. Therefore, these results support a more cautious interpretation: TM-Rephrase generally improves topic coherence, especially for LDA, BERTopic, and TSCTM, but the magnitude of effects is topic model-dependent.

#### Sensitivity Analysis Across Different Topic Numbers

As shown in Figure 4, both general and colloquial-to-formal rephrasing improved $C_{v}$ coherence across all tested K values for both models. For BERTopic, colloquial-to-formal rephrasing improved $C_{v}$ from 0.3445 to 0.3741 at 5 and from 0.4096 to 0.4682 at 20. For LDA, colloquial-to-formal rephrasing improved $C_{v}$ from 0.3102 to 0.4093 at 5 and from 0.3411 to 0.4002 at 20. These results suggest that the coherence gains from TM-Rephrase are not limited to the original 8 setting, although the magnitude of improvement varies across topic numbers and topic models.

**Figure 4.**
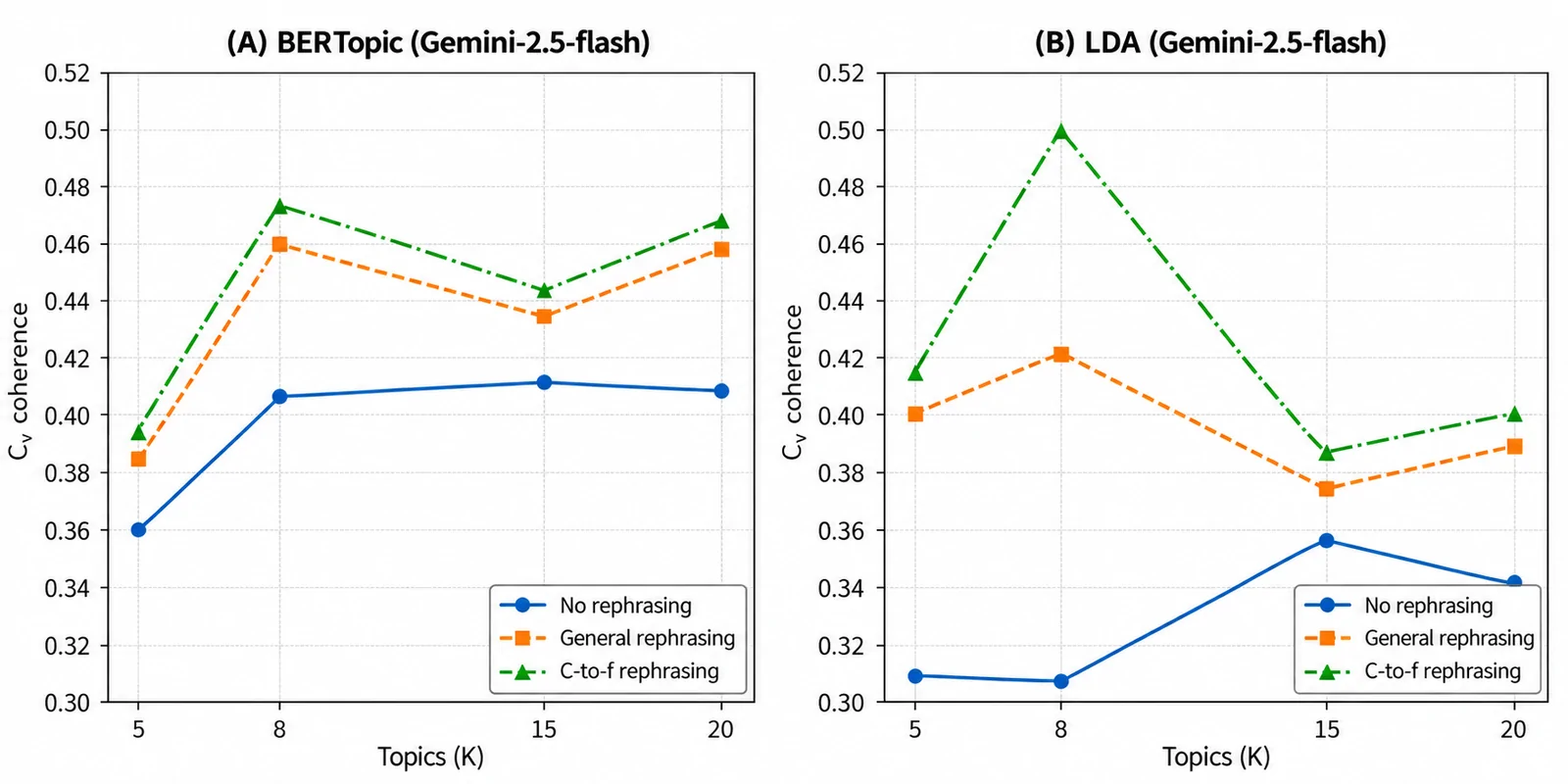
Sensitivity of topic coherence to number of topics and rephrasing schemes for (A) BERTopic and (B) LDA, based on Gemini 2.5 Flash rephrasing backbone. BERTopic: BERT-based topic modeling; LDA: latent Dirichlet allocation.

These quantitative results suggest that TM-Rephrase generally enhances intratopic coherence and can improve intertopic quality metrics for short, noisy texts in health communications, although the effects are model- and metric-dependent. The 2 different rephrasing schemes that we developed have similar overall performance. The general rephrasing scheme offers balanced and reliable improvements, while the colloquial-to-formal scheme has a slight edge, especially for lexically sensitive topic models such as LDA and BERTopic. These findings demonstrate the effectiveness and robustness of TM-Rephrase as an LLM-based model-agnostic framework for topic modeling in health communications.

### Results of Expert Ratings of Rephrasing Semantic Fidelity

The expert validation results (Table 4) provided additional evidence regarding the semantic fidelity of the 2 LLM-based rephrasing schemes. Across all 240 expert ratings, the mean semantic fidelity score was 3.90 (SD 1.01), and the median score was 4.00 (IQR 3.00-5.00) out of 5.0, suggesting that rephrasing generally preserved the original meaning.

**Table 4.** Results of expert validation on rephrasing semantic fidelity.

Validation scope	Ratings	Mean (SD)	Median (IQR)	Ratings ≥4 (%)	ICC(2,4)^a^, average-measure
Overall	240	3.90 (1.01)	4.00 (3.00-5.00)	65.42	0.736
General rephrasing	120	4.38 (0.78)	5.00 (4.00-5.00)	87.50	0.613
C-to-f^b^ rephrasing	120	3.42 (0.99)	3.00 (3.00-4.00)	43.33	0.553

a ICC: intraclass correlation coefficient.

b C-to-f: colloquial-to-formal.

However, the 2 rephrasing schemes showed different fidelity patterns. General rephrasing received higher expert ratings, with a mean score of 4.38 (SD 0.78), a median score of 5.00 (IQR 4.00-5.00), and 87.50% (105/120) of ratings of 4 or above. In contrast, the colloquial-to-formal scheme received a lower mean score of 3.42 (SD 0.99), a median score of 3.00 (IQR 3.00-4.00), and 43.33% (52/120) of ratings of 4 or above. This result suggests that general rephrasing better preserved the original meaning, stance, or intent, and tone, whereas colloquial-to-formal rephrasing introduced greater changes because of its stronger formalization.

As meaning, stance, or intent, and tone were evaluated through a single holistic rating rather than separate dimension-specific scores, the validation results cannot determine quantitatively which aspect of fidelity contributed most to the lower ratings for colloquial-to-formal rephrasing. Therefore, the lower colloquial-to-formal ratings should be interpreted as indicating a greater risk of fidelity change overall, rather than as definitive evidence that tone or stance or intent alone was the primary source of reduced fidelity. However, 1 expert noted that meaning and stance or intent were often similar while tone differed, suggesting that professionalization of tone may be one important contributor.

The overall average interrater reliability was ICC(2,4)=0.736, indicating acceptable agreement among expert raters for the aggregated semantic fidelity ratings. Scheme-specific intraclass correlation coefficient values were lower, with ICC(2,4)=0.613 for general rephrasing and ICC(2,4)=0.553 for colloquial-to-formal rephrasing. This pattern suggests that expert judgments were more variable when evaluating the degree to which formalized rephrasing preserved tone and stance, which is consistent with the greater linguistic transformation involved in the colloquial-to-formal scheme. Generally, these findings support a more nuanced interpretation: general rephrasing provides stronger preservation of the original public voice, whereas colloquial-to-formal rephrasing offers clearer formalization but involves a possible trade-off in tone and rhetorical fidelity.

### Illustrative Topic-Level Examples

To contextualize the quantitative findings, Tables 5 and 6 provide illustrative examples of topic keywords and topic assignments before and after rephrasing.

**Table 5.** Top 15 keywords for LDA^a^ topics based on Gemini 2.5 Flash (*K*=8)^b^.

Topic ID	Keywords
w/o^c^ rephrasing	
1	vaccine, covid, get, people, dont^d^, mask, kid, cdc, child, need, school, vaccinated, like^d^, work, trump
2	mask, covid, wear, virus, vaccine, wearing, people, stop, dont^d^, flu, need, face, work, social, spread
3	covid, death, vaccine, cdc, people, case, day^d^, many^d^, child, one^d^, dont,^d^ symptom, week^d^, died, year^d^
4	vaccine, effect, dose, people, covid, know, covaxin, dont^d^, pfizer, shot, like^d^, mrna, child, long, one^d^
5	covid, death, rate, case, cdc, data, infection, people, immunity, vaccination, study, show^d^, variant, state, number
6	vaccine, cdc, covid, pfizer, stop, fda, people, transmission, please^d^, health, year^d^, child, approved, public, prevent
7	covid, ivermectin, dont^d^, mask, cdc, people, work, vaccine, stop, positive, tested, like^d^, getting^d^, dose, doctor
8	vaccine, covid, vaccinated, get^d^, flu, still^d^, shot, people, child, year^d^, got^d^, even^d^, booster, fully^d^, getting^d^
w/^e^ general rephrasing scheme	
1	covid, cdc, vaccine, death, please^d^, mask, pandemic, trump, virus, could^d^, american, health, public, regarding, trust
2	mask, vaccinate, wear, covid, please^d^, personal_protective_equipment, get^d^, vaccine, school, individual, society, social_distance, virus, wearing, still^d^
3	covid, vaccine, individual, flu, vaccinated, people, case, virus, immunity, child, stop, positive, tested, get^d^, one^d^
4	vaccine, covid, ivermectin, mrna, pfizer, people, lie, individual, death, effective, health, covaxin, received, moderna, treatment
5	vaccine, covid, child, shot, booster, effective, mrna, prevent, flu, please, receive, cdc, people, covaxin, variant
6	mask, covid, day^d^, cases, test, people, virus, individual, cdc, one^d^, work, vaccine, hospital, positive, wear
7	covid, child, vaccine, death, risk, vaccination, data, year^d^, people, cdc, rate, individual, adverse, virus, cause
8	vaccine, risk, side_effect, testing, tweet, pfizer, would^d^, covid, child, vaccination, kid, also^d^, hospital, concern, original
w/ c-to-f^f^ rephrasing scheme	
1	mask, covid, individual, vaccine, public, operation, vaccinated, state, virus, health, wear, public_measure, may, work, mandate
2	covid, child, vaccine, individual, vaccination, regarding, death, concern, risk, year^d^, virus, age, variant, may, significant
3	covid, individual, vaccine, positive, test, statement, author, mask, vaccination, testing, result, current, case, information, president
4	disease, cdc, prevention, control, center, covid, tweet, ivermectin, individual, health, professional, public, treatment, travel, coronavirus
5	vaccine, covid, effect, associated, data, symptom, adverse, cdc, child, efficiency, vaccination, side_effect, potential, disease, death
6	individual, vaccine, vaccination, covid, vaccinated, regarding, may, immunity, infection, health, efficacy, influenza, public, risk, concern
7	vaccine, vaccination, covid, individual, booster, pfizer, received, pharmaceutical, dose, trust, cdc, company, fda, financial, administration
8	public, health, regarding, covid, concern, pandemic, vaccine, social_distance, significant, information, current, mask, trump, cdc, individual

a LDA: latent Dirichlet allocation.

b Results with rephrasing are based on the input texts rephrased through Gemini 2.5 Flash.

c w/o: without.

d Irrelevant keywords (off-topic words identified through author interpretation).

e w/: with.

f C-to-f: colloquial-to-formal.

**Table 6.** Illustrative examples of original, general, and c-to-f^a^ rephrased tweet replies with corresponding LDA^b^ and BERTopic^c^ topic assignments^d^.

Text type	Text content	Topic assigned by LDA	Topic assigned by BERTopic
Example 1			
Original	Are you kidding me with this? Don’t be stupid enough to let your perfect baby be a guinea pig for these people. People are getting bells palsy after getting the vaccine.	vaccine^e^, covid, get, people, dont, mask, kid^e^, cdc, child^e^, need, school, vaccinated^e^, like, work, trump	covaxin, novavax, vaccine^e^, approve, mrna, booster, ichoosecovaxin, need, want, fda, safe^e^, people, child^e^, wait, ocugen
General rephrased	Are you serious? Don’t be foolish and allow your healthy child to be a test subject for these individuals. Reports indicate people are developing Bell’s palsy after receiving the vaccine.	vaccine^e^, risk^e^, side_effect^e^, testing^e^, tweet, pfizer, would, covid, child^e^, vaccination^e^, kid^e^, also, hospital, concern^e^, original	vaccine^e^, covaxin, omicron, novavax, variant, mrna, delta, booster, approve, child^e^, vaccinate, side_effect^e^, people, risk^e^, concern^e^
C-to-f rephrased	Concerns have been raised regarding potential adverse effects, specifically Bell’s palsy, following vaccination. It is imperative to approach public health recommendations and medical interventions with informed decision-making.	vaccine^e^, covid, effect, associated, data, symptom, adverse^e^, cdc, child^e^, efficiency, vaccination^e^, side_effect^e^, potential, disease, death	vaccine^e^, covaxin, novavax, mrna, approval, subject^e^, receive, adverse^e^, booster, vaccination, risk^e^, efficacy, child^e^, side_effect^e^, concern^e^
Example 2.			
Original	Yes - let’s trust Pfizer - same company that entered a $2.3 billion criminal plea deal for lying! Same company that is exempt from liability and CANT be sued for any injuries - yup sign me right up *eye roll*	vaccine^e^, effect, dose, people, covid, know, covaxin, dont, pfizer^e^, shot, like, mrna, child, long, one	pfizer^e^, vaccine^e^, effect, mrna, covid, cdc, moderna, people, shot, child, test, get, know, stop, receive
General rephrased	Should we trust Pfizer? The same company that previously entered a $2.3 billion criminal plea deal for lying is now exempt from liability and cannot be sued for any injuries. So, yes, sign me right up. *eye roll*	vaccine^e^, covid, ivermectin, mrna, pfizer^e^, people, lie^e^, individual, death, effective, health, covaxin, received, moderna, treatment	vaccine^e^, pfizer^e^, individual, vaccination, trial, mrna, concern^e^, lie^e^, report, utility, public, moderna, cdc, fda, information
C-to-f rephrased	Concerns exist regarding public trust in Pfizer, given its history, including a $2.3 billion criminal plea deal for deceptive practices. Furthermore, the company’s exemption from liability and inability to be sued for injuries raises significant questions regarding accountability.	vaccine^e^, vaccination^e^, covid, individual, booster, pfizer^e^, received, pharmaceutical, dose, trust^e^, cdc, company^e^, fda, financial, administration	vaccine^e^, pfizer^e^, individual, vaccination^e^, responsibility^e^, mrna, concern^e^, profit^e^, business, trust^e^, public, moderna, cdc, fda, information

a C-to-f: colloquial-to-formal.

b LDA: latent Dirichlet allocation.

c BERTopic: BERT-based topic modeling.

d Results with rephrasing are based on the input texts rephrased through Gemini 2.5 Flash.

e Words that better describe topics.

These examples are intended to demonstrate how TM-Rephrase may affect interpretability. In interpreting these examples, keywords were considered more informative when they were specific, domain-relevant, and clearly aligned with public health themes, whereas generic terms, colloquial artifacts, and weakly related words were treated as less informative.

In Table 5, LDA-derived topics without rephrasing exhibit limited semantic coherence, particularly for topic 2 (public health measures), which is dominated by generic and less informative terms (eg, “mask,” “wear,” and “stop”) and includes off-topic noise such as “dont.” General rephrasing improves topical specificity by introducing more contextually meaningful health-related terms (eg, “personal_protective_equipment” and “social_distance”), although some colloquial artifacts persist (eg, “please” and “could”).

In contrast, colloquial-to-formal rephrasing produces more coherent and structured topics, characterized by standardized and policy-relevant terminology (eg, “mandate” and “public_measure”). A similar pattern is observed in vaccine-related topics (eg, topic 5), where baseline outputs contain vague terms (eg, “like” and “get”), while rephrasing, particularly for colloquial-to-formal, yields more precise and analytically meaningful keywords (eg, “adverse,” “associated,” and “efficiency”). These examples suggest that increasing linguistic formality and contextual explicitness can reduce less informative terms and make selected topic outputs easier to interpret in relation to public health discourse.

Table 6 further illustrates how rephrasing may affect the apparent semantic alignment between selected replies and their assigned topics. In Table 6, rephrasing improves the semantic alignment between tweet content and assigned topics across both LDA and BERTopic. In example 1, the original tweet expresses concerns about vaccine side effects using informal and fragmented language, resulting in generic or weakly focused topic keywords (eg, “vaccine” and “child”). General rephrasing introduces explicit health-related terms such as “side effect,” “risk,” and “concern,” improving thematic alignment. Colloquial-to-formal rephrasing further strengthens this alignment by incorporating more formal and domain-specific terminology (eg, “adverse”), enabling both models to produce more coherent and semantically grounded topics. A similar pattern is observed in example 2, where the original tweet contains colloquial and emotionally charged expressions regarding Pfizer.

Topic assignments from the original text include noisy and less informative terms (eg, “people,” “dont,” and “get”). General rephrasing improves relevance by introducing clearer semantic cues (eg, “concern”), while colloquial-to-formal rephrasing further refines the discourse with more structured and policy-relevant vocabulary (eg, “trust,” “responsibility,” and “profit”), allowing topic models, particularly BERTopic, to capture higher-level institutional themes.

## Discussion

### Principal Findings



This study developed and evaluated TM-Rephrase, a model-agnostic framework that uses LLM-based rephrasing to standardize short, informal public health texts while aiming to preserve their original meaning. Using 25,027 public replies to CDC tweets on Twitter during the COVID-19 pandemic, we found that rephrasing improved topic coherence and topic distinctiveness while generally preserving semantic fidelity across multiple topic modeling approaches.

For RQ1 (whether and how effectively TM-Rephrase improves the quality and interpretability of topic model outputs for short texts in health communications), the results show that TM-Rephrase improves topic quality and interpretability for short, noisy social media texts. The strongest coherence gains were observed for LDA, where $C_{v}$ increased from 0.3094 to 0.5004, suggesting that rephrasing helps mitigate lexical sparsity and fragmented wording. Improvements were also observed for embedding-based approaches such as BERTopic, indicating that the benefits of rephrasing are not limited to one type of topic model. Across multiple evaluation metrics, including $C_{v}$, $C_{NPMI}$, $C_{UCI}$, $U_{MASS}$, *TU*, *TR*, and *TD*, the results suggest that TM-Rephrase produces topic-word distributions that are more coherent, less redundant, and easier to interpret.

However, these improvements should be interpreted cautiously. Automated coherence metrics may partly reward the lexical regularity introduced by rephrasing, and higher topic coherence does not necessarily mean that all meaningful public-response signals are preserved. In infoveillance contexts, sarcasm, distrust, anger, uncertainty, and oppositional framing are not merely noise; they may represent important public health signals. Therefore, we interpret TM-Rephrase as improving the readability and interpretability of topic-model outputs, rather than as fully validating the substantive meaning of public responses.

For RQ2 (how different rephrasing schemes influence topic model performance), the 2 rephrasing schemes showed different effects. General rephrasing produced balanced and consistent improvements while better preserving the original public voice. In contrast, the colloquial-to-formal scheme often produced stronger gains in coherence and topic distinctiveness, especially for lexically sensitive models such as LDA and TSCTM. At the same time, colloquial-to-formal rephrasing involves greater linguistic transformation and may alter tone, rhetorical force, or framing. This suggests that prompt design should be aligned with the analytic goal: general rephrasing may be preferable when preserving public expression is important, while colloquial-to-formal rephrasing may be useful when the goal is to maximize topical clarity and model interpretability.

For RQ3 (how to interpret public discussions, that is, replies to CDC during the COVID-19 pandemic, based on the topics and inform public health practice), the improved topic representations helped clarify public responses to CDC communications. Rephrased topics more clearly captured recurring concerns related to vaccination attitudes, perceived risks, preventive behaviors, mistrust of official guidance, and uncertainty about public health recommendations. These findings suggest that TM-Rephrase can support public health social listening by helping analysts organize large volumes of noisy public replies into more interpretable thematic structures.

Practically, TM-Rephrase may serve as a human-in-the-loop preprocessing strategy for public health social listening rather than a replacement for expert interpretation. By reducing lexical fragmentation and improving the readability of topic-model outputs, it may help analysts organize large volumes of noisy public replies and identify themes that warrant closer review, such as vaccine safety concerns, mistrust of official guidance, confusion about preventive measures, or perceived gaps between public messaging and lived experience. However, this study does not directly evaluate whether TM-Rephrase improves public health decisions, communication outcomes, or detection of otherwise missed concerns. Therefore, its public health utility should be interpreted as a potential application that requires further validation in real-world infoveillance and communication workflows.

Generally, this study provides evidence that LLM-based rephrasing can enhance topic modeling of short public health texts, while also highlighting the need for careful validation. Future work should further evaluate semantic fidelity through larger-scale human expert review, topic intrusion tasks, posttopic alignment assessments, and cross-platform testing across different public health contexts.

### Limitations and Future Work

This study has several limitations that should be addressed in future research. First, during dataset curation, we did not perform additional bot or spam classification beyond the filtering described in Data Collection, because the primary objective was to evaluate the effect of rephrasing on topic modeling outputs rather than to infer public opinion. The filtering process may also introduce selection bias. In particular, replies linked to deleted or inaccessible CDC tweets were excluded, and these excluded interactions may differ systematically from the retained replies. Therefore, the final corpus should be interpreted as a dataset of public replies with recoverable CDC conversational context, rather than as a complete representation of all public interactions with CDC accounts or all COVID-19 discourse on Twitter. In addition, because the analysis focuses on replies to official CDC communications on Twitter during the COVID-19 pandemic, future research should test TM-Rephrase across other platforms, health topics, population groups, and public health communication contexts before applying it in operational surveillance or social listening systems.

Second, although LLM-based rephrasing is designed to preserve semantic meaning while improving linguistic clarity, it may introduce an important trade-off for public health infoveillance. Rephrasing can reduce lexical fragmentation and improve topic coherence, but it may also soften or alter meaningful public-discourse signals, including sarcasm, anger, distrust, misinformation cues, uncertainty, oppositional framing, and colloquial expressions. These features should not be treated merely as noise, because they may reflect public resistance, confusion, risk perception, or distrust toward health institutions.

The expert semantic-fidelity validation provides partial evidence regarding this trade-off. Overall, expert ratings suggested generally favorable preservation of meaning, stance or intent, and tone; however, general rephrasing received stronger semantic-fidelity ratings than colloquial-to-formal rephrasing. This suggests that stronger formalization may be more likely to alter tone, rhetorical force, or stance expression. Therefore, automated improvements in coherence should not be interpreted as definitive evidence of improved substantive interpretability. In infoveillance applications, original and rephrased texts should be examined together, especially when sarcasm, distrust, anger, uncertainty, misinformation cues, or oppositional framing are central to the RQ. In addition, the expert validation used a single holistic semantic-fidelity rating that asked raters to consider meaning, stance or intent, and tone together; therefore, this study cannot quantitatively determine which specific dimension of fidelity was most affected by rephrasing. Future validation should rate meaning, stance or intent, tone, uncertainty, sarcasm, and distrust as separate dimensions to better identify which aspects of public discourse are most vulnerable to meaning drift or tone normalization.

Recent AI alignment research suggests that differences across LLM backbones may extend beyond linguistic performance. Lau et al [34] found that LLMs can vary in value-priority profiles and alignment with human judgments. This is relevant to TM-Rephrase because rephrasing public discourse may involve implicit choices about tone, framing, emphasis, and socially sensitive meanings. Thus, although our results show broadly similar topic-modeling improvements across the LLMs tested, future work should examine whether different LLMs preserve meaning, stance, tone, uncertainty, and distrust in comparable ways, especially for sensitive public-health replies.

Moreover, this study used a fixed number of topics and a selected set of representative topic modeling approaches to enable consistent comparison between original and rephrased texts. Although this design supports methodological evaluation, public health communication is dynamic and context-dependent. Future work could adapt TM-Rephrase to better capture evolving public concerns across different stages of health emergencies.

### Conclusions

This study highlights the value of improving input text quality for online public health infoveillance and analytics, particularly those based on social media. By leveraging LLM-based rephrasing, TM-Rephrase enables more interpretable, consistent, and reliable detection of thematic patterns in short, noisy public responses to official health communications during health emergencies.

The data-centric and model-agnostic TM-Rephrase framework also offers a practical and scalable solution to enhance the usability of large-scale social media data for infoveillance. The findings suggest that linguistic standardization plays an important role in strengthening the interpretability of analytical outputs and supporting a clearer understanding of public concerns from large volumes of raw online data.

As social media continues to serve as a key channel for public engagement on various health topics, novel analytical frameworks, such as TM-Rephrase, enable more effective monitoring of public discourse and better-informed public health communication strategies, especially during health emergencies.

## Supplementary material

10.2196/98319 Multimedia Appendix 1 Queries, prompts, implementation details, and notice of the institutional review board exemption determination.

## Abbreviations

BERTopic: BERT-based topic modeling
CDC: Centers for Disease Control and Prevention
FASTopic: fast, adaptive, stable, and transferable topic model
ICC: intraclass correlation coefficient
LDA: latent Dirichlet allocation
LLM: large language model
NPMI: normalized pointwise mutual information
RQ: research question
TD: topic diversity
TR: topic redundancy
TSCTM: topic-semantic contrastive topic model
TU: topic uniqueness
