# Supplementary material for: Identifying Public Response Topics to the Centers for Disease Control and Prevention’s COVID-19 Communications on Social Media: Infoveillance Study Using Large Language Model–Based Rephrasing
Source: JMIR Infodemiology. 2026 Oct 8;6:e98319. doi: 10.2196/98319 (PMC13649175; doi:10.2196/98319)
Supplement: Supplementary file 1 [file infodemiology-v6-e98319-s001.pdf]

Section S1. Data Search Queries

ncov OR ncov-19 OR sars OR SARS-COV-2 OR "corona virus" OR pandemic OR pheic OR "wuhan virus" OR "china virus" OR "wuhan pneumonia" OR "wuhan flue" OR kungflue OR covid19 OR covid OR "covid 19" OR covid-19 OR coronavirus OR vaccine OR vaccines OR vaccination

Section S2. Prompt information for rephrasing schemes

Table S1. General and colloquial-to-formal prompts for TM-Rephrase

General Rephrasing Prompt
Rephrase the following tweet to improve grammar, clarity, and tone, while keeping the meaning exactly the same. Do not alter or remove any named entities, hashtags, usernames, or specific terms. Return only the rephrased tweet — no explanation, no formatting. [Original Tweet]
**C-to-F Rephrasing Prompt**
Convert the following tweet into formal, professional, and standard English suitable for inclusion in a public health report or professional summary. Preserve the original meaning and do not change or remove any named entities, hashtags, usernames, or specific terms. Avoid slang, contractions, or overly casual language. Use complete sentences and proper grammar. Return only the rephrased tweet — no explanation or formatting. [Original Tweet]

Section S3. Example tweet reply and topics assigned to it by LDA and BERTopic with and without rephrasing schemes.

Table S2. Example of original, general, and C-to-F rephrased tweet replies with corresponding LDA and BERTopic topic assignment. [a, b]

Text Type	Text Content	Topic Assigned by LDA	Topic Assigned by BERTopic
Original	Thousands attending funerals & not social distancing, thousands attending riots & not social distancing. Thousands attending protests about one thing or another & not social distancing. Thousands looting stores & businesses & not social distancing. Thousands now getting COVID	vaccine, cdc, covid, pfizer, stop, fda, people, transmission, please, health, year, child, approved, **public**, prevent	mask, wear, **people**, vaccine, covid, need, cdc, virus, work, vaccinate, stop, **social**, spread, face, **protect**
General Rephrased	Thousands are attending funerals without social distancing, and thousands are attending riots without social distancing. Thousands are attending protests on various issues without social distancing, and thousands are looting stores and businesses without social distancing. Consequently, thousands are now contracting COVID.	mask, vaccinate, wear, covid, please, **personal_protective_equipment**, get, vaccine, school, individual, society, **social_distance**, virus, wearing, still	mask, wear, vaccine, **people**, cdc, virus, individual, spread, **public**, vaccinate, **distancing**, **social**, mandate, face, **ppe**
C-to-F Rephrased	A significant number of individuals are attending funerals, riots, and protests without adhering to social distancing guidelines. Concurrently, widespread looting of stores and businesses is occurring, also without social distancing. These collective activities are contributing to a substantial increase in COVID-19 transmission.	**public**, health, regarding, covid, **concern**, pandemic, vaccine, **social_distance**, significant, information, current, mask, trump, cdc, individual	mask, wear, individual, **public**, health, mandate, **measure**, transmission, vaccination, **distancing**, **ppe**, **protection**, **concern**, efficacy, **social**

[a]Words that can better describe topics are **in bold** and highlighted.
[b]Results with rephrasing are based on the input texts rephrased through Gemini-2.5-flash.

In Table S2, the original tweet discusses public gatherings and COVID-19 risks using repetitive and loosely structured phrasing. Topic keywords derived from the original text are semantic-fragmented and quite unrelated (e.g., *"Pfizer"*, *"work"*, and *"fda"*). Under general rephrasing and C-to-F rephrasing, newly introduced terms such as *"personal protective equipment", "ppe"*,

"social distancing", and *"distancing"* significantly enhance semantic specificity, resulting in more coherent topic assignments. These newly surfaced terms (highlighted in the table) enable both LDA and BERTopic to converge on well-defined themes related to personal protection and social protective measures.

Section S4. Implementation Details

For LLM-based rephrasing, we used prompt-based generation with three LLM backbones, i.e., Gemini-2.5-Flash, GPT-4o-mini, and Mistral-7B-Instruct-v0.2. GPT-4o-mini was accessed through the OpenAI API, Gemini was accessed through the Google generative AI API, and Mistral-7B-Instruct-v0.2 was run locally in a quantized format. The rephrasing scripts used low-temperature decoding where generation parameters were explicitly specified, with temperature set to 0.2 for GPT-4o-mini and Mistral-7B. Mistral was run with top_p = 0.95 and repeat_penalty = 1.05.

For text preprocessing, we used regular expressions, NLTK, and spaCy. URLs, punctuation, user mentions, and nonalphabetic characters were removed where applicable. Text was converted to lowercase, tokenized, and lemmatized. For the NLTK-based pipeline, tokenization used word_tokenize, lemmatization used WordNetLemmatizer, and stop words were removed using the NLTK English stopword list. Tokens shorter than three characters were removed. For the BERTopic-style topic representation step, spaCy en_core_web_sm was used for tokenization and lemmatization, with stop words, punctuation, nonalphabetic tokens, and one-character lemmas removed.

For the main topic modeling experiments, the number of topics was fixed at $K = 8$, and the top 15 keywords were extracted for each topic. In the sensitivity analysis, K was varied across 5, 8, 15, and 20 for LDA and BERTopic. LDA was implemented using Gensim LdaMulticore. The dictionary was constructed from the preprocessed corpus and filtered using no_below = 5 and no_above = 0.5. LDA was trained with passes = 15, chunksize = 100, alpha = "asymmetric," eta = "auto," per_word_topics = True, and random_state = 100.

The BERTopic-style pipeline used SentenceTransformer embeddings with all-MiniLM-L6-v2. Document embeddings were reduced using UMAP with n_neighbors = 15, n_components = 5, metric = "cosine," low_memory = True, and random_state = 0. Because a fixed number of topics was required for direct comparison across conditions, KMeans was used for clustering with n_clusters = K, random_state = 0, and n_init = "auto." Topic representations were generated using class-based TF-IDF with CountVectorizer and a spaCy-based lemmatizing tokenizer.

FASTopic was implemented using the FASTopic package with the TopMost Preprocess module. The main setting used num_topics = 8, DT_alpha = 10.0, and the same top-15 keyword extraction strategy. Random seeds were set using torch.manual_seed(0) and np.random.seed(0) before model training. TSCTM was implemented using the TopMost TSCTM module. The TSCTM configuration used vocab_size = 5000, en_units = 200, temperature = 0.5,

weight_contrast = 1.0, epochs = 20, batch_size = 32, and learning_rate = 0.002, with torch and NumPy random seeds set to 0.

Topic coherence was computed using Gensim CoherenceModel. In addition to C_v, we calculated C_{NMPI}, C_{UCI}, and U_{MASS} to assess whether the observed improvement patterns were dependent on a single coherence metric. When external reference texts were required for coherence calculation, English Wikipedia text was used as the reference corpus. Topic uniqueness, topic redundancy, and topic diversity were calculated from the top keyword lists of the generated topics.

Section S5. Notice of Determination of Exemption from IRB Office in UNC Charlotte

CHARLOTTE
DIVISION OF RESEARCH

**To:**	Wangjiaxuan Xin<br>Public Hlth & Hlth Admin
**From:**	Office of Research Protections and Integrity
**Approval Date:**	08-Jul-2026
**RE:**	Notice of Determination of Exemption
**Exemption Category:**	2
**Study #:**	IRB-26-1225
**Study Title:**	Human Evaluation of Topic Modeling Performance for Text Analysis of Public Health-Related Social Media Short Texts

This submission has been reviewed by the Office of Research Protections and Integrity (ORPI) and was determined to meet the Exempt category cited above under 45 CFR 46.104(d). This determination has no expiration or end date and is not subject to an annual continuing review. However, you are required to obtain approval for all changes to any aspect of this study before the changes can be implemented and to comply with the Investigator Responsibilities detailed below.

Your approved consent forms (if applicable) and other approved documents are available online at Submission Page.

Investigator's Responsibilities:

1. Use **only** the approved versions of study materials (e.g., recruitment scripts, consent forms, data collection materials, etc.).
2. Amendments **must** be submitted for review and the amendment approved before implementing the changes. This includes changes to study procedures, study materials, study personnel, etc.
3. Be aware that this study is included in the **Post-Approval Monitoring program** and may be selected for post-review monitoring at some point in the future.
4. Researchers must adhere to all site-specific requirements mandated by the study site (e.g., access requirements and/or restrictions, etc.).
5. Data security procedures must follow procedures as approved in the protocol application and/or data security plan in accordance with OneIT Guidelines for Data Handling.
6. Promptly notify the IRB office (uncc-irb@charlotte.edu) of any adverse events or unanticipated risks to participants or others. You may be required to submit an Adverse Event form in Niner Research as well.
7. Five years (5) following this approval/determination, you must complete the **Administrative Check-in** form via Niner Research to provide a study status update.
8. Failure to submit the Administrative Check-in will result in a process hold on future submissions until the administrative check-in is complete.
9. Complete the **Closure eform** via Niner Research once the study is complete.
10. Faculty Advisors are responsible for ensuring student PIs fulfill these Investigator Responsibilities.

Please be aware that approval may still be required from other relevant authorities or "gatekeepers" (e.g., school principals, facility directors, organization leadership, listserv administrators, custodians of records, etc.).

Figure S1. Proof of IRB Exemption Approval Determination
